# Automated Cell Identification and Tracking Using Nanoparticle Moving-Light-Displays

**DOI:** 10.1371/journal.pone.0040835

**Published:** 2012-07-19

**Authors:** James A. Tonkin, Paul Rees, Martyn R. Brown, Rachel J. Errington, Paul J. Smith, Sally C. Chappell, Huw D. Summers

**Affiliations:** 1 Centre for Nanohealth, Swansea University, Singleton Park, Swansea, United Kingdom; 2 School of Medicine, Cardiff University, Heath Park Cardiff, United Kingdom; Emory University/Georgia Insititute of Technology, United States of America

## Abstract

An automated technique for the identification, tracking and analysis of biological cells is presented. It is based on the use of nanoparticles, enclosed within intra-cellular vesicles, to produce clusters of discrete, point-like fluorescent, light sources within the cells. Computational analysis of these light ensembles in successive time frames of a movie sequence, using k-means clustering and particle tracking algorithms, provides robust and automated discrimination of live cells and their motion and a quantitative measure of their proliferation. This approach is a cytometric version of the *moving light display* technique which is widely used for analyzing the biological motion of humans and animals. We use the endocytosis of CdTe/ZnS, core-shell quantum dots to produce the light displays within an A549, epithelial, lung cancer cell line, using time-lapse imaging with frame acquisition every 5 minutes over a 40 hour time period. The nanoparticle moving light displays provide simultaneous collection of cell motility data, resolution of mitotic traversal dynamics and identification of familial relationships allowing construction of multi-parameter lineage trees.

## Introduction

Computerized identification, discrimination and tracking of biological cells, in microscopy images, is vital to modern high throughput platforms that deliver automated scanning and capture of millions of images per day [1]–[3]. Rapid, machine-based image analysis is now essential as the data generation rate far exceeds human processing capacity and many of the key challenges in cell biology demand knowledge of all individuals within large cell populations, e.g. understanding the role of heterogeneity and division asymmetry in cancer [4]–[7] or stem cell proliferation and differentiation [8]. Through the use of ever-increasing processing speed and capacity and evolving microscopy techniques, automated cell identification and spatio-temporal tracking is now widely used [9]–[11]; however it is far from straightforward to implement and requires computational algorithms and imaging science beyond that common to standard microscopy. Thresholding and segmentation routines used to identify cell outlines are often complex, reflecting the intrinsic problem of poor optical contrast within epi-illuminated or bright-field images, caused by the minimal refractive index differences between cells and their surrounding environment. Phase contrast or fluorescence imaging modalities alleviate some of these problems [12]–[13] but have varying applicability across cell-types due to changing optical density in the case of phase-based techniques or necessitate intervention in the cell biology to introduce fluorescence markers, e.g. GFP transfection, antibody loading or DNA staining; this can interfere with natural cell function and so application to live cells is limited [14]. Even when successful cellular image analysis has been implemented there often remains a fundamental imbalance between data acquired and information processed: large data-set images are taken at sub-cellular resolution and then processed to produce much simpler, whole cell parameters such as cell identity, type, position etc. This is in-efficient processing of information and imposes an overhead on hardware performance, computational power and data analysis time.

These computerized approaches mimic human visual perception of form and motion where dense and complex image information is processed to obtain much simpler, abstract representations of objects and their position. However, through early studies by Wertheimer and others on the relationship between perception and simplified abstractions, such as points or lines, it is now known that human perception can operate directly at the level of the abstract object and so does not require detailed information – the human form of a ‘stick-person’ is recognizable despite consisting only of straight lines and a circle. This is the *Gestalt* (“unified whole”) theory of visual perception [15] and its consequence to image analysis is that acquisition need not incorporate the full spatial detail of the object. This realization was put to practical use in the early 1970’s by Johansson who utilized our ability to accurately discriminate and track objects with minimized information by studying human motion using moving light displays (MLD), created from video sequences of high contrast optical sources attached to the joints of a moving person or animal [16]. The technique has been widely adopted in the computer image community and is now routinely used for optical motion capture and animation through imaging of dark suited actors with bright optical sources or reflectors, positioned at key points, which describe the mechanics of movement [17]–[19]. In the context of imaging cytometry the MLD technique demonstrates that accurate identification and quantification of cell motion does not require high spatial resolution of cellular form and structure and can be performed with a low number of binary optical markers associated with the cell.

**Figure 1:**
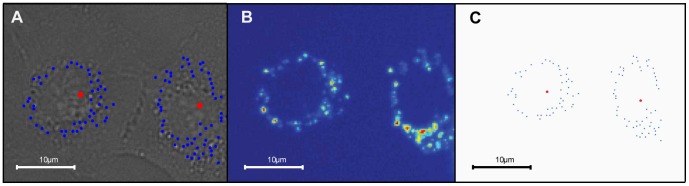
Typical cell images. (A) Bright field image with binary element overlay; (B) fluorescence image of two neighboring A549 cells; (C) the light point cluster map derived from the fluorescent signal. The binary elements within the light point map are displayed as blue points and represent the locations of the centres of the quantum dot labeled vesicles; the centroids of the binary element clusters are displayed as red points. (see Movie S1 for mitosis animation).

In this paper we report on the implementation of the moving-light-display technique in cells by the use of lysosome-encapsulated quantum dots (QDs) to create clustered points of light, through which cells can be identified, tracked and analyzed. The creation of the optical sources through endocytosis of fluorescent markers provides a generic and innate encoding mechanism, applicable across multiple cell types. The nanoparticles provide robust, bio-stable and photo-stable fluorescence that can be tracked over multiple generations, and at nanomolar concentrations do not perturb cellular function [20]. Under typical loading conditions there are between 10–50 vesicular light sources; these are resolvable at low magnification and provide good signal to noise discrimination due to particle concentration within the vesicles. A typical panel of images of quantum dot labeled cells is shown in figure 1; this depicts the acquired bright-field (1A) and fluorescence (1B) images and a binary map created from the fluorescence data (1C, see materials and methods for details of the image processing). The fluorescently-labeled lysosomes are observed as clusters of light sources, the cellular encapsulation of which provides a localization of the cluster to the intra-cellular domain; this is sufficient to discriminate between clusters and hence identify individual cells. Cell movement is analyzed through identification of the cluster centroid and tracking of its spatial translation in successive time frames. The use of point tracking in biological image analysis is well established [21]–[23]; here we are adapting the approach to obtain integrative information at the level of the cell rather than the individual points. As in human MLD the light points allow recognition of the form and motion of the gross object (the cell) through a reduced information set of 10–50 binary-valued pixels (the cluster centroids). The inter-relationship of light sources within a cluster is determined by the relative motion of the labeled lysosomes and hence it carries biological information relating to cellular state and function. For example, the local-level metric of mean point separation within a cluster provides a quantitative measure of a cell’s traversal through mitosis with associated spatial arrangements of the QD-loaded lysosomes providing specific, easily recognizable geometrical motifs of cell division.

(details presented in section on *analysis of cell division*).

In the following sections a demonstration of the moving-light-display concept within cells is presented; starting with details of the microscopy and image analysis techniques used and then expanded through reference to the biologist’s requirement for i. identification and discrimination of cells, ii. spatio-temporal tracking of their motion and division, iii. analysis of division events and iv. visualization of time dependent relationships in cell lineage maps. We conclude with a discussion on the applicability of the technique and a general summary of the nanoparticle MLD approach.

## Materials and Methods

### Cell Culture

A549 (ATCC CCL-185) cells were maintained under G418 selection in McCoy’s 5a medium supplemented with 10% fetal calf serum (FCS), 1 mM glutamine, and antibiotics and incubated at 37°C in an atmosphere of 5% CO_2_ in air. For imaging experiments, cells were grown at a density of 1×10^6^ cells ml^−1^ as a monolayer in either coverglass bottomed chambers (Nunc, 2 Well Lab-Tek II, Fisher Scientific) or glass bottomed (24 multi-well Sensoplate, Greiner Bio-one for 24 h prior to imaging. All cell concentrations were determined using a Coulter Particle Counter (Beckman Coulter, High Wycombe, UK).

### Nanoparticle Loading

Cells were loaded with commercially available targeted nanocrystals using the Qtracker® 705 (QTracker705) Cell Labeling Kit (Invitrogen (Q25061MP) at 4 nM concentration. The reagents in the Qtracker® 705 Cell Labeling Kit use a custom targeting peptide (9-arginine peptide) to deliver near-infrared-fluorescent nanocrystals into the cytoplasm of live cells via the endosomal pathway. Briefly, Qtracker reagent A and B were premixed and then incubated for 5 minutes at room temperature. 1 ml of fresh full growth media was added to the tube and vortexed for 30 seconds. This labeling solution was then added to each well of the cells and incubated for 1 hour at 37°C after which they were washed twice with fresh media. Subsequently 24 hours later, labeled cells were then analyzed by time-lapse, confocal microscopy.

**Figure 2:**
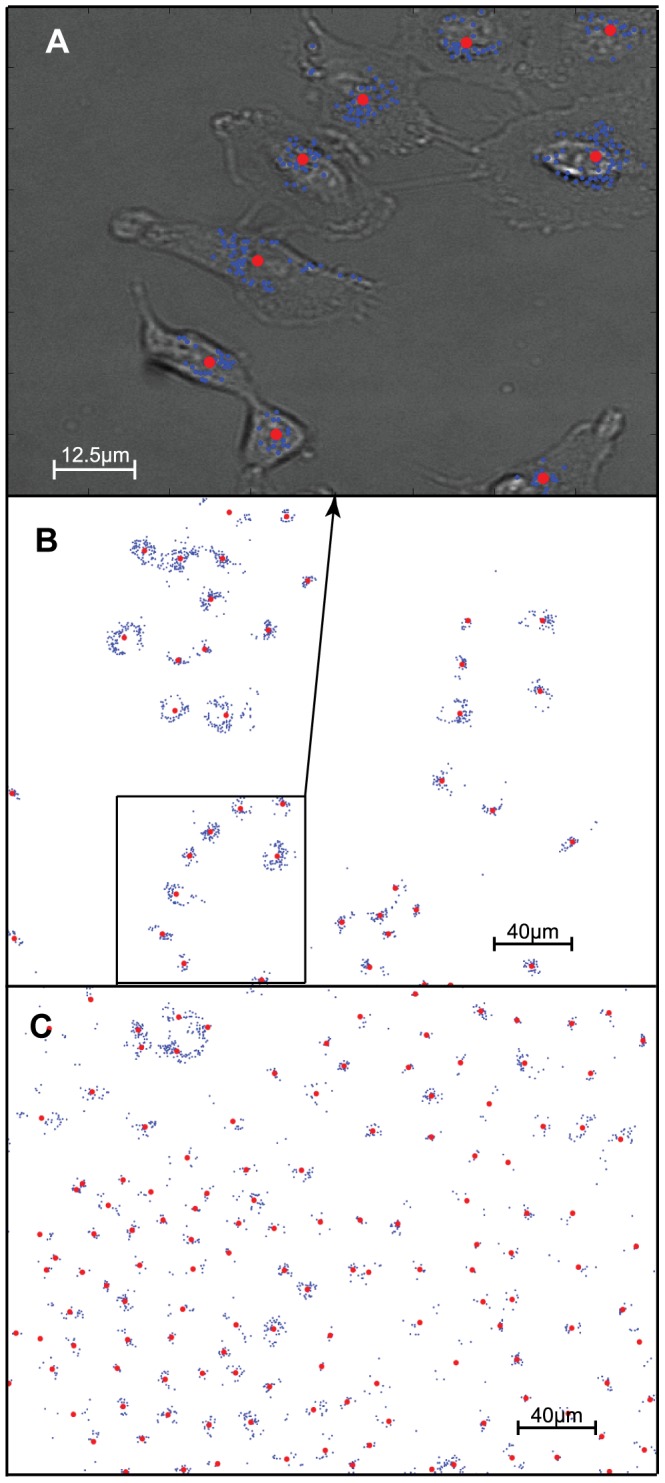
Spatio-temporal cell tracking. (A) Bright field image of A549 cells overlaid with binary elements (blue) and assigned centroids (red); (B) full-field binary element representation of a 336×256 µm fluorescence image taken at the initial time point of experiment and (C) representation of the same field at the t = 40 hour time point. (see Movie S2 for evolution animation).

### Image Acquisition

Confocal laser scanning microscopy (Radiance CLSM, BioRad Ltd) was used to track quantum dot labeled A549 cells over a 48 hour period. The Qtracker705 fluorescence was collected using 488 nm excitation and 680–20 nm emission filters; x,y,z,t optical sections (using ×40, 0.75 NA air lens) were collected every 5 minutes.

### Image Pre-processing

All image processing was done within the MATLAB programming environment. The fluorescence image data was provided in the format of multi-layer tiff stacks each containing 8 focal plane images for every 5 minute interval across the 48 hour experimental range. The capture of multiple focal planes allowed creation of a composite image using the highest contrast regions of all available images. This composite was created by segmenting the image space into smaller regions and applying the standard absolute gradient algorithm of the form:
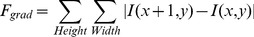
(1)where I(x,y) is the intensity of the pixel (x,y).

Conversion to a binary representation is a simple two step process.

- A step function of the form
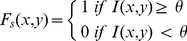
(2)where θ is a pixel intensity threshold defined as
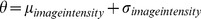
(3)was applied to the composite fluorescence image to remove background noise. Here µ is the image intensity average and σ the standard deviation added to account for variability of background.
- A simple peak finder algorithm locating the maximum pixel intensity within a localized area by cross-referencing the linear profiles of the pixel rows and columns was applied to locate the maximum intensity points of the fluorescent signals corresponding to nanoparticle loaded vesicles and their x and y coordinates stored.

**Figure 3:**
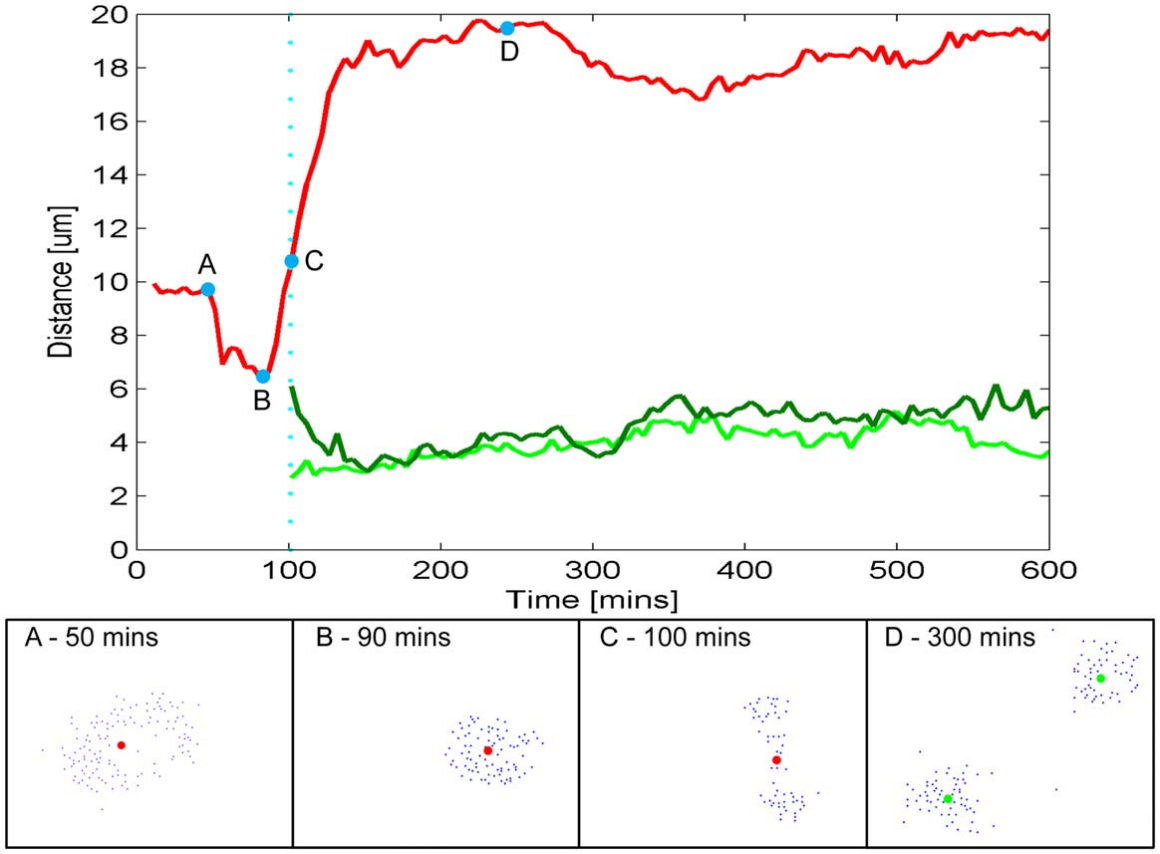
Analysis of mitosis. (main figure) An example of the characteristic curve of mean binary element separation as an individual cell undergoes a mitotic event and divides into two daughters cells. (sub-panels) The corresponding stills below show the binary element distribution, in a fixed frame position, at four time points spanning key stages of the process: (A) Cell prior to extracellular signs of mitotic committal; (B) localized contraction of the light-point markers as the cell prepares to divide; (C) marker distribution indicating telophase stage; (D) two daughter cells identified as independent clusters (frame scale is 40×30 µm). (see Movie S3 for animation).

### Cluster Analysis – the First Pass

A standard k-means clustering analysis algorithm was used to identify groups of pixels in the binary image (binary elements) corresponding to fluorescent vesicles within the same cell. For the binary elements coordinates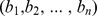 we iteratively used the function:
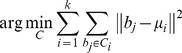
(4)to find the cluster centroid location. Here 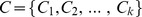 is the set of clusters with total cluster number k, b_j_ is the set of binary elements associated with the cluster C_i_ and µ_i_ is the mean of the binary points in **C**_i_. The seeding of the centroid locations in the initial timeframe was done manually and the k-means algorithm then applied to refine their coordinates to the true cluster centers. The in-built k-means algorithms within Matlab proved to be accurate provided the initial seed centroid locations were accurately assigned by the user.

In successive images the centroid locations from the previous timeframe were used as a seeding set. To deal with the sporadic occurrence of rogue binary elements (noise) and binary elements representing cells entering the field of view, new centroids are assigned to regions containing binary elements that are more than 120 pixels (30 µm or ∼3 cell diameters) from their nearest centroid. New seeds are assigned to deal with both these occurrences as they are indistinguishable without time consuming comparisons with previous frames and interpretation of boundary events. It is temporally and computationally more efficient to assign binary elements to new clusters generated according to basic rules and interpret the nature of the groupings during later processes. A proximity validation was applied by identifying current seeds with no binary elements within a distance of 50 pixels (12.5 µm) and removing them. This primarily deals with the event of a cluster moving out of the field of view therefore leaving a centroid seed from the previous frame with no binary elements to define it and secondarily with centroids previously defined to account for rogue elements whose intensities have dropped back below the noise filter. The k-means algorithm was then run using the modified centroid set and a measure of the cluster fit taken by implementing a native Matlab silhouetting algorithm to acquire a cluster fit parameter. The Silhouette process provides a validation of the clustering by determining how well each binary element fits within its assigned cluster. For each binary element *i*, let a_i_ be a measure of dissimilarity with elements in the same cluster, in our case the average distance from all other cluster members. Perform the same operation comparing element *i* with all elements in all other clusters successively and assign the lowest dissimilarity measure as b_i_. Now define the silhouette of the binary element *i* as
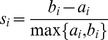
(5)where 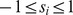. A value close to 1 means the element fits well with its assigned cluster and the average s_i_ of the all binary elements is a measure of how well assigned clusters are. Following optimal assignment of cluster centroids an average binary element separation parameter was calculated for each cluster sequentially. If this parameter was found to be greater than 15 µm, ∼1.5 average cell diameters, it was likely that a division event had occurred and so the k-means algorithm is run again with an additional centroid seed placed 10 pixels from the centroids associated with probable division events. The silhouette cluster fit parameter was then re-calculated and if the value found to be more favorable the additional centroid is accepted. The process was reiterated until all clusters were validated.

### Cluster Refinement – Centroid Linkage and the Mitotic Signature

Refinement of the spatial tracking was accomplished by temporal tracking of the centroids and by identification of cell division events through further analysis of the separation of binary elements within clusters.

The centroids in each time frame were linked to centroids in surrounding time frames through application of a nearest neighbor map. For two sequential frames with centroid sets **C**_i_ and **C**_i+1_ where 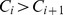
we calculated the Euclidian distance between each member of **C**_i_ and **C**_i+1_ and then assigned nearest neighbors in ascending order until each centroid of **C**_i+1_ was linked to a corresponding centroid in **C**_i_. The remaining centroids in **C**_i_ were then linked to their respective closest centroids in **C**_i+1_ provided they are within the average single cell diameter of 10 µm otherwise they were deemed to be new progenitor groupings. The centroid lineages were then stored as vectors of length |**C**_i_| whose entries are the indices of the corresponding closest centroid in **C**_i+1_. Division events revealed themselves at this point when a single centroid in one frame had two nearest neighbors in the next. With lineages initially defined the temporal placement of division events was further refined by tracking each of the lineages individually through all timeframes and compiling mean distance profiles to search for the characteristic cluster contraction and expansion dynamic that is a signature of cell mitosis.

(see *analysis of cell division*).

**Figure 4:**
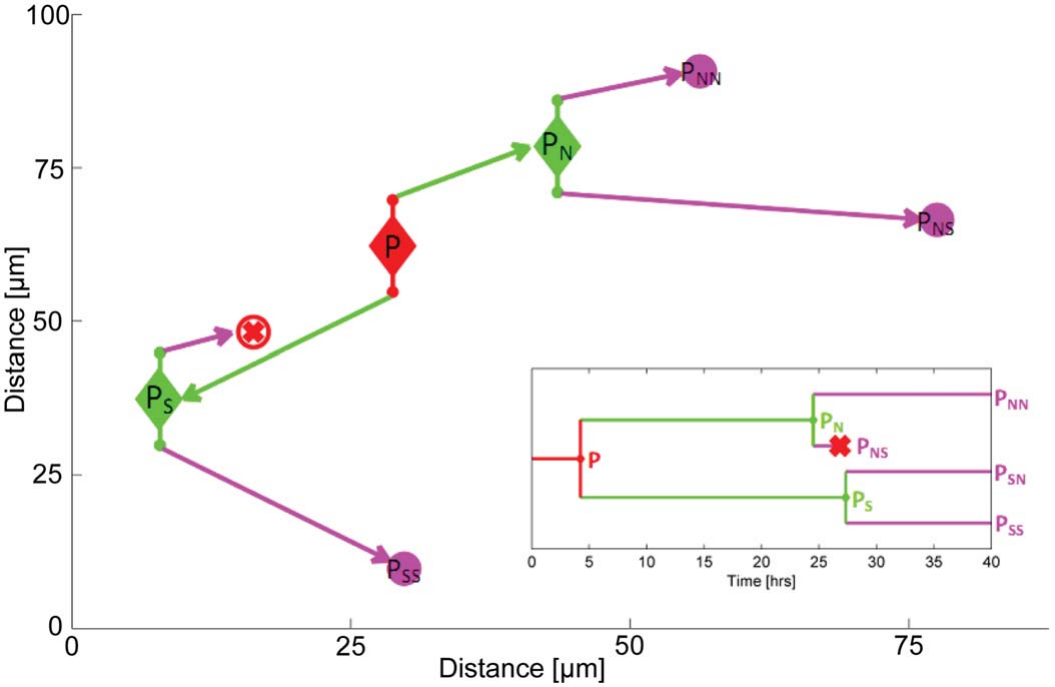
Lineages. Graphical representation of a single cell’s lineage evolution through space alongside conventional lineage portrait as defined by the automated centroid tracking. Diamonds mark the spatial and temporal location of mitosis events, red being the progenitor cell, green the second generation cells and magenta the location of the cell centroids in the final frame of the time-lapse sequence. The cross marks the loss of a cell behind an unidentified object in the image (non-cell). The arrows in the main diagram may be viewed as motility vectors reporting the mean velocity of a cell between mitotic events.

## Results

### Cell Identification

A typical example of the embedding of point light sources within cells, through the use of QDs, is shown in figure 1. The nanoparticle loaded vesicles form a perinuclear ring of punctate fluorescence (fig. 1B). It is the location of the vesicles rather than the overall light distribution that we require. The fluorescence image is therefore filtered to identify points of peak intensity which are then used to create a binary image, in which, discrete pixels form a digital map of points (fig. 1C) representative of the nanoparticle vesicles (see materials and methods section for full detail). We refer to these point sources as ‘binary elements’. It is the spatial relations and temporal motion of the binary elements that provide identification of cells and analysis of their motion, function and proliferation. The localization of nanoparticle loaded vesicles within individual cells produces clustering of the binary elements which can be recognized through a k-means clustering analysis, and represented by, a centroid marker which corresponds to the geometrical centre of the binary elements (fig. 1C). Thus the complex graphical information of the bright field image (fig. 1A) is reduced to a set of Cartesian co-ordinates (the centroid markers), each of which represents a cell, and through which the spatio-temporal behavior of the cell population is analyzed. Previous studies have shown the QTracker705 QDs to be photo-stable over many days and that the QD loaded lysosomes are conserved upon cell division [24]. Identified cells can therefore be tracked across the cell cycle and the proliferating population mapped through detection of division events and the associated daughter cells.

### Spatio-temporal Tracking of Cells

Once an initial field of cells has been identified and cluster centroids assigned, a full spatio-temporal track can be obtained through linkage of the centroids through successive time frames. Centroid seeds for each successive frame are defined to optimize the speed of the k-means process, account for the possibility of losing and gaining clusters off the edge of the plane of view and deal with rogue binary element points occurring sporadically through time in otherwise empty regions. The general approach taken can be summarized as follows: 1. generate a foundation set using centroid locations from the previous time frame, 2. validate proximity of all centroids to binary elements to identify centroids whose binary element cluster has moved out of view and 3. validate proximity of binary elements to centroids to account for any noise elements that passed through the filter. Images were taken with a 5 minute time interval; this minimizes the probability of large changes in cell position and thus ensures accurate correlation of centroid locations from frame to frame.

A movie sequence of this centroid motion and the ‘birth’ of new centroids in daughter cells, taken over a 48 hr period, is shown in Movie S2; single images, taken at the 0 and 40 hr time points, are shown in figure 2. A composite, overlay image shows the relation of the QD-encoded binary elements to the cluster centroid and to the detailed topology of the cell, as seen in the bright field image (figure 2A). The A549 cell-line has a mean inter-mitotic time of ∼22 hours [25] and so there are 2–3 rounds of cellular division during the time of the experiment. The images in figure 2B and 2C clearly show this proliferation, the centroid number increases from 41 to 133 and the average number of binary elements per cluster reduces from 34 to 12. These cluster statistics are consistent with a cell doubling (QD vesicle number halving) time of ∼23 hours.

**Figure 5:**
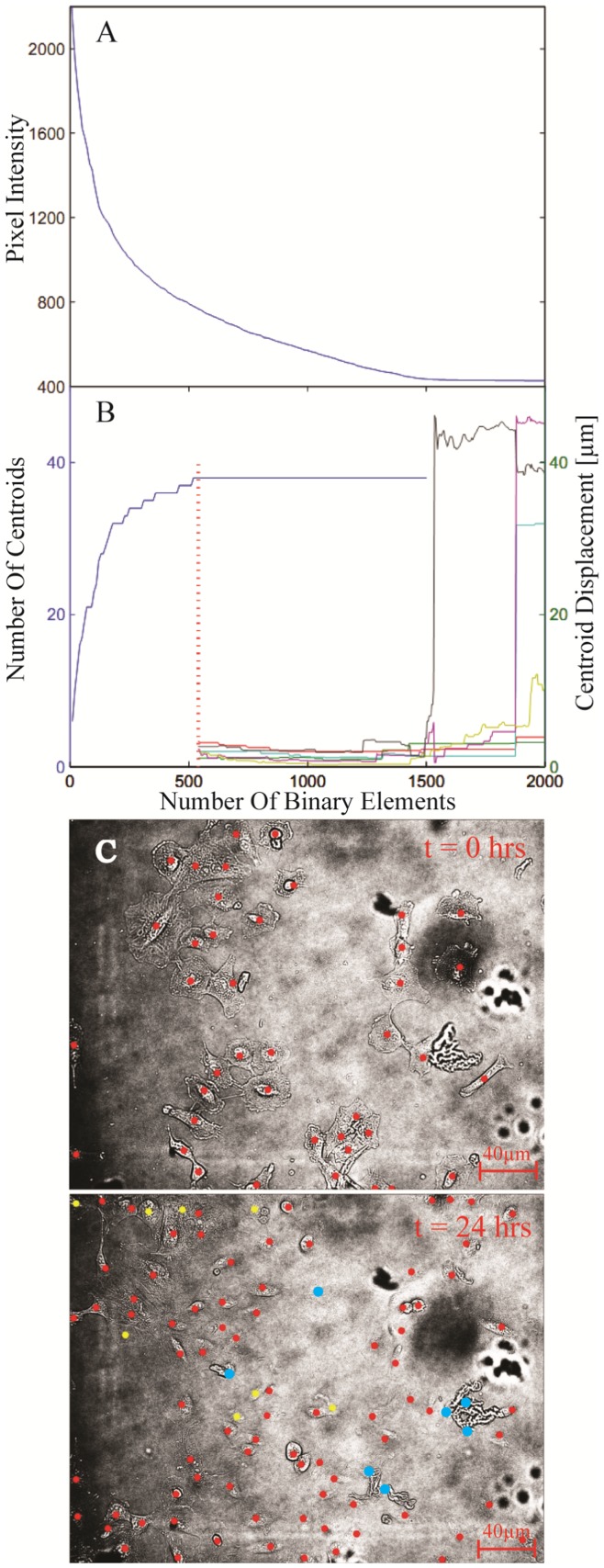
Operational range. (A) Calibration curve of initial image showing the relation between the intensity threshold filter cut-off and the number of binary elements identified. (see Movie S4 for animation) (B) Plot showing the operational range of the system through the binary element dependence of the number of centroids (cells) identified (black curve), and the centroid displacement of a selection of cells (colored curves). The vertical, red dashed line indicates the minimum point number requirement for maximization of the number of cells automatically identified. (C) Bright field images at t = 0 and t = 24 hours with centroids overlaid. Red spots indicate automatically identified centroids, blue spots correspond to incorrectly assigned centroids and yellow spots represent manually identified cells which are unidentifiable by the automated analysis. (see Figure S1 for panel 3 in higher resolution).

### Analysis of Cell Division

As cells go through mitosis they undergo distinct morphological changes that can be tracked via the binary elements. The mean distance of the binary elements from each cell centroid provides a characteristic parameter, from which a ‘mitotic curve’ may be constructed (figure 3). As cells enter the mitotic phase they detach from the adherent surface and round-up to a spherical geometry, this condenses the nanoparticle vesicle distribution and produces a reduction in the mean binary element separation (fig. 3A–3B). After chromosomal separation the nanoparticle vesicles disperse during cell telophase and the mitotic curve begins to increase (fig. 3B–3C). Upon the completion of mitosis the binary element spacing continues to grow as two clusters separate in the daughter cells (fig. 3C–3D); this latter increase arises from the computational assignment of elements to a single centroid whereas physically there are clearly two separate groupings following cellular division. We therefore make a transition from a single to a pair of centroids and reassign the binary elements to daughter cells when the average separation of a cluster after the contraction phase surpasses a predefined level, defined as the stable separation distance maintained prior to contraction (fig. 3C).

The mitotic curve provides not only a clear digital marker of cell division events but also a quantitative, analogue track through the mitotic phases which informs on the kinetics of the cell division process (see supporting media for further examples). Here the moving point display technique provides a tool with which the shape and motion of cells can be analyzed. The linkage of cell division events through spatio-temporal tracking provides a functional lineage analysis capable of describing the clonal relationships of a wide range of morphological and motility measures. As an example, a motility lineage map is shown in figure 4 in which the inter-generational relationships are mapped by velocity vectors, displaying the mean speed and net direction of inter-mitotic cellular motion.

### Accuracy and Robustness

In order to numerically quantify the operational range of the nanoparticle-encoded MLD we take a representative sample image and investigate the effects on cluster identification and assignment of centroid position due to variation in the noise filter threshold (figure 5). This approach is chosen from a wide range of alternatives as it directly relates the accuracy of the technique to the number of encoding light points (nanoparticle loaded vesicles) and corresponds to the experimental measurable of fluorescence signal to noise (SNR). Increasing the intensity of the thresholding step function reduces the number of binary elements identified, as noisy, rogue pixels are filtered out (fig. 5A). From the conversion plot in fig. 5A we calculate the number of cluster centroids identified and the displacement of a selection of these, as a function of the total number of binary elements within the image frame (fig. 5B). There is an operational range between ∼500–1500 elements over which the technique provides accurate identification of cell identity and position independent of the QD signal SNR. The accurate identification of cells is determined by the number of binary elements available to define a cell cluster. Using the initial timeframe for this operational range quantification, it was found that 520 binary elements were needed for all 38, manually identified, cluster centroids to have at least 1 binary element assigned to them (the red dashed line in fig. 4B). This corresponded to a noise intensity cut-off of 784 units (SNR = 2.8) and is a ‘worst case’ in that sufficient binary elements had to be defined to locate all centroids. Relaxing this criterion still identifies the majority of centroids by a minimum of 5 binary elements (30 centroids located from 200 binary pixels) using a threshold cut-off of 1100 (SNR = 2). The minimum number of elements necessary to identify a cell is an important criterion in studying proliferating cells as the QD loaded vesicles will be diluted upon division [24]. Over the 40 hour time series shown in figure 2 the mean cluster number per cell reduces to 12, we would anticipate therefore that 72 hours of tracking could be achieved (3–4 cell divisions) before over-dilution makes cell identification impossible.

If the noise threshold filter is reduced too far the binary element number begins to relate to background noise rather than valid QD encoding pixels. Whilst the presence of these noisy pixels does not immediately invalidate the identification of cells (a random noise source adds pixels to each cell cluster with equal probability) it does affect the centroid co-ordinates and so leads to inaccurate determination of cell position. As the threshold filter approaches the noise floor (intensity ∼ 400 units) and the total number of binary elements increase beyond 1500 the centroid positions show marked deviations in excess of average cell diameters (>10 µm). However SNR values as low as 1.1 can be tolerated before this noise weighting produces a cluster position offset of 10 µm.

To assess the accuracy of centroid seeding (cell identification) and spatio-temporal tracking two image frames corresponding to the 0 and 24 hour time points were chosen. An initial centroid set was chosen at the intial timepoint by visual inspection of the bright field image; the number of centroids in the timeframe captured 24 hours later was then automatically assessed using the MLD algorithms and compared to direct visual identification. The 24 hour imageframe is shown in figure 5C with automatically identified centroids overlaid on the bright field image; 81% of manually identified cells are correctly mapped.

## Discussion

Quantum dots are widely used for cellular labeling as they provide both a photo and bio stable fluorescence marker that can be spectrally tuned. Here we show that the processing of these nanoparticles by the cell is as important as their innate photonic properties; they are naturally taken up and concentrated into vesicles that are dispersed throughout the cytoplasm and as such provide bright, point like light sources across the majority of the cell cytoplasm. This bio-processing thus enables image analysis by these discrete points which are the fundamental elements of a moving light display. Concentration on representative sub-sampling of the image rather than full visualisation has two major benefits: i. major data reduction and ii. access to rapid and efficient image examination based on cluster analysis techniques. The processing of a bright field image to produce a binary map of QD light-points (fig. 1A–C) leads to a reduction in data size from ∼2.5 MB to 20 kB per image. In high-throughput applications, where standard acquisition may produce 1 TB of data per day, there is a pressing need for such data reduction to avoid the ever increasing penalty in cost and time of data analysis. Throughout this work we have knowingly adopted simple algorithms to demonstrate the robustness of the moving light display approach and to highlight the advantage of undertaking cluster based analysis. Further development of these algorithms can only increase the accuracy of tracking and cellular event interpretation. For example, cell definition in terms of marker clarity and resolution significantly degraded towards the edge of the field of view and the addition of pre-processing routines on the original raw fluorescence image to account for the lens effects and variability in background noise across the image would help in enhancing these regions as would the application of more sophisticated noise filtering. The critical limiting factor of the MLD technique is cell identification as cells become increasingly confluent and harder to resolve without knowledge of their respective boundaries. One possible solution to increase the ability to resolve individuals at high confluency is to apply a nuclear marker at the end of the experiment. Using the final nuclear stained cells as the starting point and running a time reversed analysis would provide a well defined initial seeding of centroids and makes identification of the bifurcation points within a lineage far simpler due to the centroids of the daughter cells converging to their progenitor thus removing the decision of when the binary elements groups are best described as dual rather than singular clusters.

To summarise, we have used quantum dot nano-particles as point like markers within a cell to demonstrate the concept of moving light display to resolve cellular mitosis events and lineages without the need for complex interpretation of a complete visual picture of the cellular field. In the study of cell movement and proliferation the knowledge we seek can be resolved at the whole cell level and so the reduction of the data set to a minimal set of Cartesian co-ordinates provides a much greater efficiency of information processing, distilling the experimental measurements down to match just that needed to understand the biology. In future applications this approach will allow real-time data processing during image acquisition and the direct storage of biological knowledge, i.e. cell position and familial relationships.

## Supporting Information

Figure S1 **Additional for** **Figure 5** **, Panel C.** An enlargement of Panel C provided in higher resolution for clarity.
(TIF)

Movie S1 **Multi-format mitosis event.** An example of a mitosis event displayed using bright field microcopy images, fluorescence microscopy images and a moving light display (MLD) representation of the binary elements and their respective centroid.
(WMV)

Movie S2 **Binary element evolution.** A sample movie showing the cellular evolution displayed through bright field microscopy and a visualization of the binary elements over a 40 hour period.
(WMV)

Movie S3 **Characteristic curve of a mitosis event.** A mitosis event can be identified by tracking the average separation of the binary elements associated with a designated group. As a cell contracts to a low energy state it becomes circular which draws the QDot markers towards the centre causing their average separation to decrease (blue line). This reaches a minimum prior to the cell dividing at which point the binary elements are best described as two unique clusters corresponding to the daughter cells (magenta and green lines). Mapping the separation of binary elements as a single cluster throughout (blue line) shows an infeasible single cell distance due to the daughter cells migrating apart.
(WMV)

Movie S4 **Pseudo-noise filter.** Investigation of the effects of increased noise is possible by varying the cut-off of the noise filter during the image cleaning process. The graphic demonstrates the effects of this variation on a single time frame. The blue dots correspond to binary elements, the red dots correspond to the centroids identified through clustering of the binary elements and the green dots mark the manually identified locations of the cells. As the filter is lowered the binary element clusters increase to sufficient numbers for each cell to allow accurate centroid allocations. As the filter approaches the noise floor of the image an increasing number of noise peaks are wrongly identified as binary element markers and these randomly occurring features result in the miss-allocation of centroids.
(WMV)
