# Supplementary figures and images for: Automated Cell Identification and Tracking Using Nanoparticle Moving-Light-Displays
Source: PLoS One. 2012 Jul 19;7(7):e40835. doi: 10.1371/journal.pone.0040835 (PMC3400648; doi:10.1371/journal.pone.0040835)

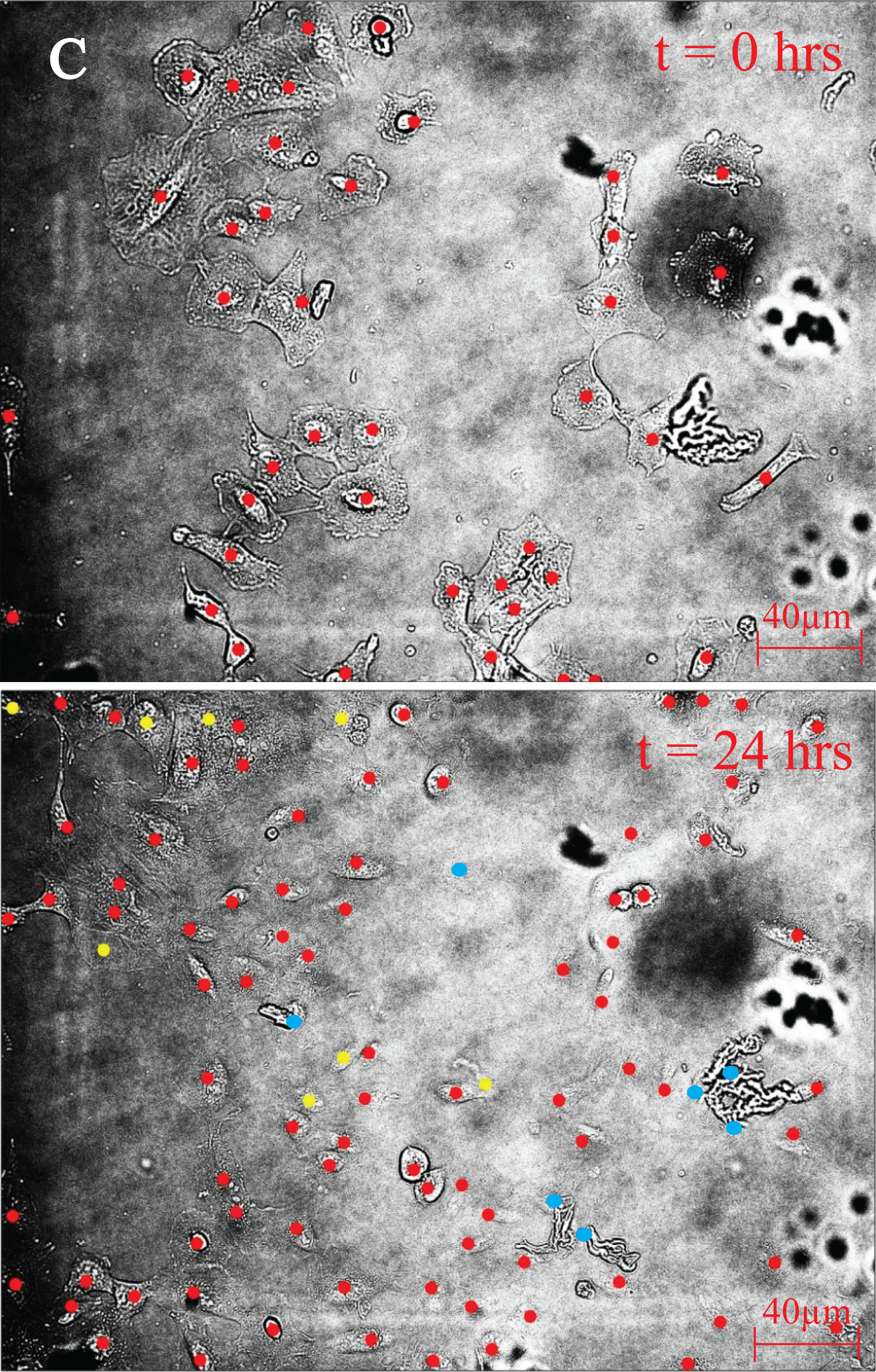

Supplement: Figure S1 — Additional for Figure 5 , Panel C. An enlargement of Panel C provided in higher resolution for clarity. (TIF) [file pone.0040835.s001.tif]
